# Getting Started in Computational Mass Spectrometry–Based Proteomics

**DOI:** 10.1371/journal.pcbi.1000366

**Published:** 2009-05-29

**Authors:** Olga Vitek

**Affiliations:** Departments of Statistics and Computer Science, Purdue University, West Lafayette, Indiana, United States of America; Princeton University, United States of America

## Introduction

*Proteomics* aims at a large-scale characterization of localization, abundance, post-translational modifications, and biomolecular interactions of the proteins in an organism, with the goal of understanding their function. An extensive insight can be obtained by identifying and quantifying the components of biological mixtures. For example, a) In studies of biomolecular networks, partners interacting with a protein can help determine its function. It is possible to experimentally isolate protein complexes, e.g., using tag affinity purification. Identification of the components of this mixture helps determine potential interactors [1]. b) Post-translational modifications such as phosphorylation play an important role in regulating biological processes, e.g., cellular growth and signaling. Identification and quantification of phosphorylated proteins and their substrates helps elucidate complex signaling pathway phosphorylation events [2]. c) Molecular biomarkers, i.e., proteins for which changes in abundance are indicative of an early onset of a disease or a therapy response, are of interest in clinical research. Identifying and quantifying components of a biofluid such as serum helps detect proteins with such discriminative ability [3]. d) A goal of genome annotation is the discovery and validation of protein-coding regions. Identifying peptides and proteins in a cell helps confirm and improve the annotations at the translational level, e.g., by confirming the presence of intron boundaries or alternative splicings [4].

*Mass spectrometry* is a method of choice for protein identification and quantification due to its sensitivity and to the versatility of the instrumentation [5],[6]. A typical “bottom-up” workflow experimentally digests the proteins into a mixture of peptides with an enzyme such as trypsin. This is necessary, in part, because the sensitivity of the mass spectrometer is much higher for peptides than for proteins. The peptides are then injected onto a liquid chromatography (LC) column from which they elute sequentially. The eluted peptides are ionized and separated by the mass spectrometer according to their ratio of mass to charge (*m/z*) in a mass spectrum (MS).

**Figure 1:**
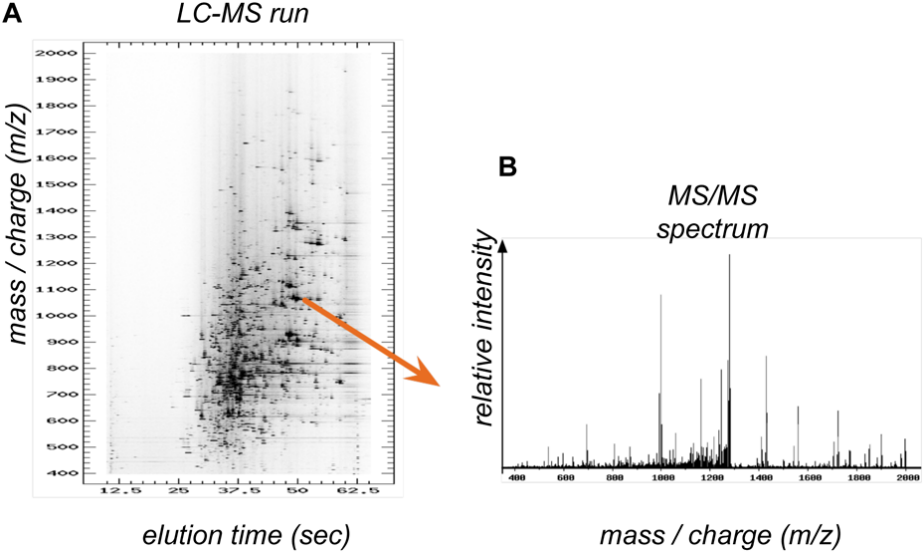
Example of spectral data. (A) LC-MS run. Features in the LC-MS space are peptide ions; their intensity is related to peptide abundance. (B) MS/MS spectrum. The spectrum is obtained by fragmenting the peptide ion isolated from an LC-MS peak. The peaks are fragment ions; distances between peaks are used for peptide sequence determination.

The collection of mass spectra obtained at different elution times forms an LC-MS run shown in Figure 1A. Peaks in the run correspond to peptide ions; however, the sequence of amino acids underlying each peak is unknown. For identification, the mass spectrometer isolates the biological material from a peak (called precursor ion in this context), and subjects it to a high-collision energy. The energy breaks the peptide at different amide bonds, and the resulting fragments are separated according to their *m/z* in a secondary spectrum (called MS2, MS/MS, or tandem MS), shown in Figure 1B. Distances between peaks in the MS/MS spectrum are used to infer the peptide sequence of the parent LC-MS peak.

Peak intensity is related to the abundances of peptides, and can be used for relative quantification. With the label-free approach, a separate LC-MS run is obtained for each biological sample, and peaks are quantified and compared across runs. In stable isotopic labeling workflow, samples from different groups are labeled metabolically (e.g., in SILAC, where stable isotopes are included in the growth medium of an organism), or chemically (e.g., in ICAT or iTRAQ, where reacting chemical labels are applied after tryptic digestion). Several samples (e.g., one from each group) are then mixed, and their peaks are identified and quantified within the same run. Finally, a targeted workflow based, for example, on selected reaction monitoring (SRM) [7], increases sensitivity and specificity by monitoring signals from a list of predefined peptides.

The design of proteomic experiments, and subsequent analysis of the spectra, involves extensive computation and requires expertise at the intersection of computer science, engineering, and statistics. It presents exciting opportunities for both methodological and applied computational research.

## Experimental Design

Experimental design specifies how biological samples are selected and allocated in space and time during spectral acquisition. For example, a biomarker discovery project can produce biased conclusions if patients from different groups have different characteristics (such as prior medication), or their spectra are acquired under different conditions. Moreover, sample selection and allocation can be inefficient, and can undermine the ability to uncover the true differences between groups.

Statistical experimental design avoids bias and optimizes efficiency by using replication, randomization, and blocking, and by choosing an appropriate type and number of replicates [8]. The need for a statistical design of proteomic experiments is increasingly emphasized [9]. Specific choices require a statistical model that describes the spectra, and development of such models is an important area of research.

## Open Data Formats

After spectral acquisition, the first computational task is to extract and store peak information. Unfortunately, most mass spectrometer vendors have their own proprietary formats. An advance has been made by implementing open XML-based formats (such as mzXML), and the associated converters and validators, to store this information and to make the subsequent analysis vendor-neutral [10]. These tools are available from http://www.proteomecommons.org. Efforts are invested, for example, by the Proteomics Standards Initiative (http://www.psidev.info), in further developments of XML formats.

## Identification of Peptides and Proteins

**Figure 2:**
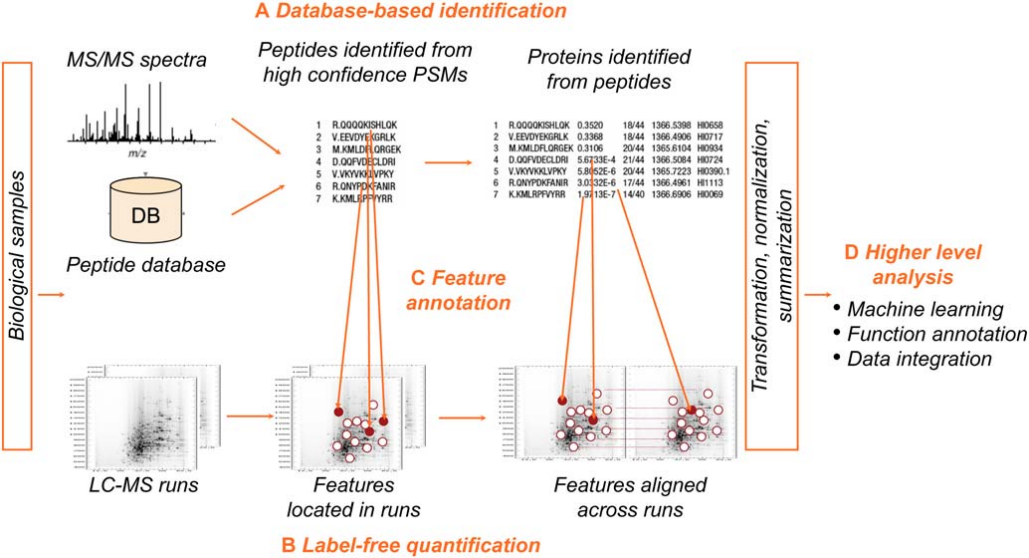
Example of a proteomic workflow using database-based identification and label-free quantification. (A) Identification of MS/MS spectra. Experimental spectra are compared to peptides in a database, and the best-scoring PSMs are reported while controlling the FDR. Protein sequences are identified from the peptides. (B) Label-free quantification. Features in LC-MS runs (shown with circles) are located, quantified, and aligned across runs. (C) LC-MS features are annotated with peptide sequences when identifications are available (shown with filled circles). The annotations are used to optimize the alignment of features across runs. The list of quantified, identified, and aligned features is then subjected to transformation, normalization, and summarization. (D) The list of features is used as input to machine learning, functional annotation, and data integration steps.

An MS/MS spectrum such as in Figure 1A is generated by a series of peptide fragments. Thus, mass differences between neighboring MS/MS peaks are used to determine the underlying amino acid sequence. Typical approaches involve searches of an a priori–defined database, de novo identifications, and combinations of the two [11]. Here we focus on the database-based approach which compares each observed spectrum against entries in a database (Figure 2A). Several aspects of the procedure require consideration.

### Database of Candidate Peptides

Protein sequence databases now exist for many organisms. One can digest the sequences in silico into peptides, and construct a theoretical spectrum for each peptide. Alternatively, one can use a library of peptides with associated consensus experimental spectra derived from previous identifications [12]. In both cases, the number of candidate peptides increases exponentially when we allow nonspecific enzymes and/or post-translational modifications (PTM) that alter a theoretical mass.

### Scoring Function

Scoring functions quantify the similarity of a candidate peptide-spectrum match (PSM). A typical two-stage procedure filters out PSMs with incompatible peptide and precursor ion masses, and scores plausible PSMs using counts of shared MS/MS peaks. Newer scores incorporate additional characteristics, e.g., peak intensity (for spectral libraries) and empirical peptide detectability [13], and learn the scores dynamically from the data [14],[15].

### Search Algorithm

For each observed spectrum, the algorithm scores its similarity to every candidate peptide and returns the best-scoring PSM. Since typical experiments produce hundreds of thousands of MS/MS spectra, development of efficient search algorithms is an active area of research. Improvements include clustering the observed spectra using a similarity metric, and only searching the resulting consensus spectra [16]. Another approach aligns the observed spectra in a procedure similar to genomic sequence alignment, and creates meta-spectra that cover longer protein segments [17]. Finally, a de novo identification of short sequence tags (e.g., three amino acids long) combined with a subsequent database search also allows one to reduce the space [18].

### False Discovery Rate (FDR) of Spectral Identification

Due to the stochastic variation in the spectra, deficiencies of the scoring schemes, and possible incompleteness of the database, only a fraction of best-scoring PSMs are typically correct. There is thus a need for a statistical measure of “confidence” in a reported list of PSMs, and for an inferential procedure that distinguishes “confident” PSMs from noise.

An accepted statistical measure is FDR, defined as the expected proportion of incorrect identifications in a list of PSMs with scores above a cutoff. To determine FDR-controlled lists of PSMs, the target–decoy strategy [19] appends a randomized version of the theoretical database (decoy) to the actual database (target), and estimates the FDR as twice the proportion of decoy matches among all the matches in the list. Alternatively, Peptide Prophet [20] fits an Empirical Bayes two-group mixture model to scores of correct and incorrect identifications, and estimates the FDR as the fitted probability of correct identifications for scores above a cutoff. Numerous extensions are continually proposed (see, e.g., http://pubs.acs.org/toc/jprobs/7/1), and in the future the focus will likely broaden to the FDR of peptides, proteins, and protein sites.

### Protein Inference

Confidently identified peptides can be grouped to infer the protein components of the mixture. This is nontrivial due to ambiguous mappings of peptides to proteins, and to the insufficient discrimination of some proteins by the identified peptides [21]. Current approaches use characteristics such as the number of mapped peptides, protein length, and peptide detectability [22] to identify proteins. More research is needed to control the FDR in the protein list.

### Resources

Extensive spectral databases are publicly available, e.g., the Peptide Atlas at http://www.peptideatlas.org, containing millions of spectra from biological experiments, and http://regis-web.systemsbiology.net/PublicDatasets, containing spectra from controlled protein mixtures.

## Quantification

Quantitative proteomics monitors peptide and protein abundance across samples of multiple types. The goals are similar to other high-throughput experiments such as gene expression microarrays [23],[24]. A typical workflow (Figure 2) involves multiple steps [25].

### Signal Processing

Quantitative workflows require signal processing beyond spectral identification. Features in the spectra must be located and quantified, annotated when possible with peptide sequences information, and aligned across runs. A variety of tools have been implemented [26]; they are specific to label-free or labeling workflows, but all output a list of detected features and their abundances across samples.

### Transformation, Normalization, and Summarization

The biological effects are multiplicative in nature, and a logarithm transform of intensities is frequently recommended. Feature intensities are further normalized across runs, e.g., using quantile normalization [27]. When multiple features are observed within a sample for a same peptide or protein, they are often summarized in one number.

### Learning

Statistical and machine learning tools are then applied for (1) *class comparison*, e.g., determination of proteins that change in abundance between healthy individuals and individuals with disease; (2) *class discovery*, e.g., unsupervised detection of sample subgroups with homogeneous quantitative protein profiles; and (3) *class prediction*, e.g., a supervised prediction of a disease status of a new sample based on its protein abundance. Here analysis issues are similar to, e.g., gene expression microarrays, in that the features are interdependent, and their number exceeds the number of samples. An example from this area of research is [28].

### Functional Annotation

Database technologies connect the proteins to their annotations, e.g., from Gene Ontology, or from databases of disease. The annotations can confirm the plausibility of the identifications, and can enable tests for over-represented functional categories in the protein list [29].

### Data Integration

Recent studies combine proteomic measurements with gene expression and metabolomic profiles, and/or known biochemical networks, with the general goal of protein function determination [30]. A number of tools facilitate these tasks, which include proprietary databases GeneGo and Ingenuity, and open-source Cytoscape at http://www.cytoscape.org and Bioconductor at http://www.bioconductor.org.
